# Addition of Immune Checkpoint Inhibitor to Platinum Retreatment for Recurrent Non‐Small Cell Lung Cancer After Perioperative Chemotherapy: A Multicenter Retrospective Study

**DOI:** 10.1111/1759-7714.70269

**Published:** 2026-03-30

**Authors:** Toshiaki Takakura, Ryota Shibaki, Atsushi Washioka, Yusuke Murakami, Yuhei Harutani, Hiroaki Akamatsu, Nobuyuki Yamamoto

**Affiliations:** ^1^ Internal Medicine III Wakayama Medical University Wakayama Wakayama Japan; ^2^ Department of Pulmonary Medicine Hashimoto Municipal Hospital Hashimoto Wakayama Japan; ^3^ Department of Respiratory Medicine NHO Minami Wakayama Medical Center Tanabe Wakayama Japan; ^4^ Department of Respiratory Medicine NHO Wakayama Medical Hospital Hidaka‐gun Wakayama Japan; ^5^ Department of Respiratory Medicine Naga Municipal Hospital Kinokawa Wakayama Japan

**Keywords:** immune checkpoint inhibitor, non‐small cell lung cancer, platinum‐based chemotherapy, postoperative recurrence

## Abstract

**Introduction:**

The addition of immune checkpoint inhibitor (ICI) to platinum‐based chemotherapy has improved outcomes in patients with advanced non‐small cell lung cancer (NSCLC). However, evidence on the efficacy of adding ICI to platinum retreatment in patients who relapse after perioperative platinum‐based chemotherapy remains limited.

**Methods:**

We retrospectively analyzed patients with recurrent NSCLC following perioperative platinum‐based chemotherapy. Patients were categorized into either the “chemo group,” who received platinum retreatment without ICI, or the “ICI‐chemo group,” who received platinum retreatment with ICI. The primary endpoint was progression‐free survival in patients treated with ICI.

**Results:**

Among 124 patients who received perioperative platinum therapy and surgery, 31 underwent platinum retreatment: 17 in the chemo group and 14 in the ICI‐chemo group. PFS was significantly longer in the ICI‐chemo group than in the chemo group (15.4 vs. 5.9 months; log‐rank *p* = 0.023; HR 0.40, 95% CI, 0.18–0.90). Among patients with PD‐L1 ≥ 50%, the ICI‐chemo group showed a greater trend toward longer PFS compared with the chemo group (16.9 vs. 4.0 months; HR 0.11, 95% CI, 0.01–1.05).

**Conclusions:**

Adding ICI to platinum retreatment may be an effective option for patients with NSCLC who relapse after perioperative chemotherapy, particularly in those with PD‐L1 expression ≥ 50%.

## Graphical abstract

We retrospectively analyzed 31 patients with NSCLC treated with platinum retreatment who relapsed after perioperative platinum therapy and surgery. PFS was significantly longer in the ICI‐chemo group than in the chemo group. Adding ICI to platinum retreatment may be an effective option for this population.

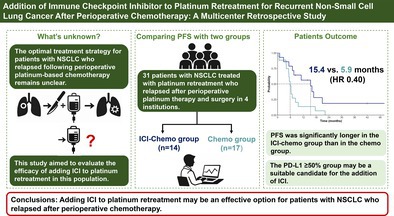

## 1 Introduction

Lung cancer remains a leading cause of cancer‐related mortality worldwide [1]. Approximately 20%–25% of patients diagnosed with non‐small cell lung cancer (NSCLC) present with resectable disease [2]. Perioperative chemotherapy is the standard of care for early‐stage NSCLC, but its benefit in reducing disease recurrence is modest, with a 5‐year recurrence rate of approximately 40%–60% [3, 4]. Development of effective treatments for patients with recurrent NSCLC following perioperative chemotherapy is therefore desired.

For patients with recurrent NSCLC, platinum retreatment has been considered the standard of care [5, 6, 7]. In contrast, in advanced‐stage NSCLC, the addition of immune checkpoint inhibitor (ICI) to platinum‐based chemotherapy has been shown to improve both progression‐free survival (PFS) and overall survival (OS). Notably, ICI has made long‐term responses possible in advanced NSCLC; for example, the 5‐year duration of response was approximately 20% among patients treated with platinum‐based chemotherapy combined with ICI [8, 9]. These findings represent a paradigm shift in the treatment strategy for advanced NSCLC.

Patients with a lower tumor burden may especially derive benefit from ICI [10]. Indeed, ICI has demonstrated remarkable efficacy in patients with early‐stage NSCLC [11]. Furthermore, patients with recurrent NSCLC typically exhibit a relatively low tumor burden [12]. ICI is thus anticipated to be effective in patients with postoperative recurrent NSCLC. In KEYNOTE‐189 and KEYNOTE‐407 studies, patients with postoperative recurrence who received perioperative therapy were eligible if the therapy was completed at least 12 months before the development of metastatic disease, and at least 6 months before randomization in the IMpower‐150 study, respectively [13, 14, 15]. However, the proportion of patients receiving perioperative chemotherapy in the ICI arm of the KEYNOTE‐189 and KEYNOTE‐407 studies was only 7.3% and 1.8%, respectively. Furthermore, subgroup analysis results for this patient population have not been published. Furthermore, in the IMpower‐150 study, the proportion of patients receiving perioperative chemotherapy is not even known. Therefore, the efficacy of platinum‐based chemotherapy with ICI for postoperative recurrence remains unclear from these Phase III studies. This study therefore evaluates the efficacy of adding ICI to platinum retreatment in patients who relapsed following perioperative platinum‐based chemotherapy.

## 2 Materials and Methods

### 2.1 Study Design and Patients

This retrospective cohort study was conducted across four institutions. Clinical data were collected from electronic medical records of patients who met the following eligibility criteria: pathologically confirmed NSCLC; treatment with perioperative platinum‐based chemotherapy and radical surgery; postoperative recurrence between January 1, 2014 and December 31, 2023; and receipt of platinum‐based retreatment with or without ICI as any line. Local radiotherapy was permitted for oligometastasis. Platinum retreatment was defined as the administration of at least one cycle of a platinum‐based regimen. However, we excluded patients who received platinum retreatment in combination with either sequential or concurrent radiotherapy.

Patients were categorized into two groups: those treated with platinum retreatment without ICI (“chemo group”), and those treated with platinum retreatment in combination with ICI (“ICI‐chemo group”). Data were collected up to the cutoff date of June 30, 2024.

This study was conducted in accordance with the principles of the Declaration of Helsinki and it was approved by the Wakayama Medical University Ethics Committee (approval number: 4408).

### 2.2 Study Objectives and Endpoints

The primary objective was to evaluate PFS in patients who received platinum‐based retreatment combined with ICI. Treatment outcomes were assessed using the Response Evaluation Criteria in Solid Tumors (RECIST) version 1.1. PFS was defined as the time from the initiation of platinum retreatment to the date of confirmed disease progression, and OS was defined as the time from the initiation of platinum retreatment to death from any cause or the date of last follow‐up.

### 2.3 Statistical Analysis

Baseline patient demographics and clinical characteristics were summarized using descriptive statistics. Comparisons between the chemo and ICI‐chemo groups were made using Fisher's exact test for categorical variables and the Wilcoxon rank‐sum test for continuous variables. Kaplan–Meier survival curves were constructed for both PFS and OS, and comparisons between the groups were made using the log‐rank test.

An exploratory analysis was conducted to identify factors associated with the efficacy of ICI‐chemo, using Cox proportional hazards models. Variables with a *p* value ≤ 0.2 in univariate analysis were included in the multivariate analysis, given the limited sample size. Statistical significance was defined as a two‐sided *p* value of < 0.05. All analyses were performed using R software, version 4.4.2.

## 3 Results

### 3.1 Patient Characteristics

**FIGURE 1:**
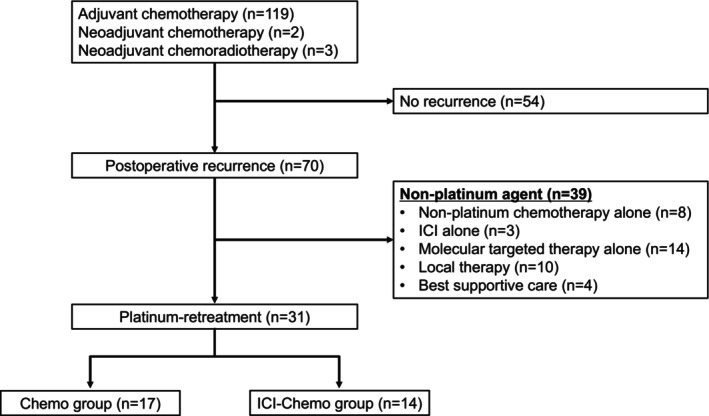
Flowchart of patients included in the study. ICI, immune checkpoint inhibitor; chemo, chemotherapy.

Among the 124 patients who underwent perioperative platinum therapy and radical surgery, 31 received platinum retreatment; 17 in the chemo group and 14 in the ICI‐chemo group, as postrelapse treatment (Figure 1). The median age of the patients was 68 (range, 47–79) years. Most patients were male (77%) and current or former smokers (77%). Regarding pathological stage, 14 (45%) and 17 (55%) were classified as stage II and III, respectively. Among the patients, 22 (71%) had nonsquamous cell carcinoma and nine (29%) had squamous cell carcinoma. Programmed death‐ligand 1 (PD‐L1) expression was assessed using the 22C3 pharmDx assay in all cases. PD‐L1 evaluation was performed using preoperative bronchoscopic biopsy specimens in 10 patients, surgical specimens in 15 patients, and rebiopsy specimens at recurrence in one patient. Although rebiopsy after recurrence was performed in nine patients, PD‐L1 re‐evaluation was conducted in only one case, and the PD‐L1 result was identical between the surgical specimen and the rebiopsy specimen. PD‐L1 expression levels of ≥ 50%, < 50%, and unknown were observed in eight (26%), 18 (58%), and five (16%) patients, respectively. All patients received a combination of cisplatin and vinorelbine as adjuvant chemotherapy. None of the patients underwent preoperative ICI. Eight patients received local radiotherapy prior to platinum retreatment. No significant differences were observed between the two groups (Table 1). The platinum retreatment regimens are summarized in Table S1. In five patients, platinum retreatment was discontinued within three cycles due to adverse events or deterioration of performance status, whereas all remaining patients completed four or more cycles. Platinum retreatment was initiated in the first, second, third, and fourth quarters in 1, 6, 11, and 13 patients, respectively. At the data cutoff, four patients were still receiving maintenance therapy following platinum retreatment. Seventeen patients received subsequent systemic therapy after platinum retreatment, whereas 10 patients died or received best supportive care without further treatment. The postplatinum retreatment systemic therapies included cytotoxic chemotherapy in 16 patients, ICI monotherapy in two patients, and molecular targeted therapy in one patient. The median number of subsequent treatment regimens was one (range, 0–4).

**TABLE 1:** Patient characteristics.

	All (*n* = 31)	Chemo (*n* = 17)	ICI‐chemo (*n* = 14)	*p*
Age				> 0.99
Median, range (years)	68 (47–79)	69 (47–79)	68 (49–75)	
≥ 65 years, *n* (%)	23 (74)	13 (81)	10 (67)	
Sex, *n* (%)				> 0.99
Male	24 (77)	13 (81)	11 (79)	
Female	7 (23)	4 (19)	3 (21)	
ECOG‐PS, *n* (%)				0.72
0	15 (48)	9 (53)	6 (43)	
1	16 (52)	8 (47)	8 (57)	
Smoking history, *n* (%)				0.41
Yes	24 (77)	12 (71)	12 (86)	
No	7 (23)	5 (29)	2 (14)	
Histology, *n* (%)				0.46
Nonsquamous cell carcinoma	22 (71)	11 (65)	11 (79)	
Squamous cell carcinoma	9 (29)	6 (35)	3 (21)	
EGFR/ALK, *n* (%)				0.13
Positive	9 (29)	7 (41)	2 (14)	
Negative or unknown	22 (71)	10 (59)	12 (86)	
PD‐L1 expression, *n* (%)				0.68
≥ 50%	8 (31)	3 (25)	5 (36)	
< 50%	18 (69)	9 (75)	9 (64)	
Pathological stage, *n* (%)				0.29
II	14 (45)	6 (35)	8 (57)	
III	17 (55)	11 (65)	6 (43)	
Type of recurrence, *n* (%)				> 0.99
Local recurrence	8 (26)	4 (24)	4 (29)	
Distant recurrence	23 (74)	13 (76)	10 (71)	
Treatment line of platinum retreatment as the cytotoxic chemo, *n* (%)				
1	30 (97)	16 (94)	14 (100)	> 0.99
≥ 2	1 (3)	1 (6)	0 (0)	
Platinum‐free interval				
Median, range (months)	20 (3.3–77)	17 (3.3–68)	20 (4.1–77)	0.95

Abbreviations: ALK, anaplastic lymphoma kinase; ECOG‐PS, Eastern Cooperative Oncology Group performance status; EGFR, epidermal growth factor receptor; PD‐L1 programmed cell death‐ligand 1.

### 3.2 Efficacy

**FIGURE 2:**
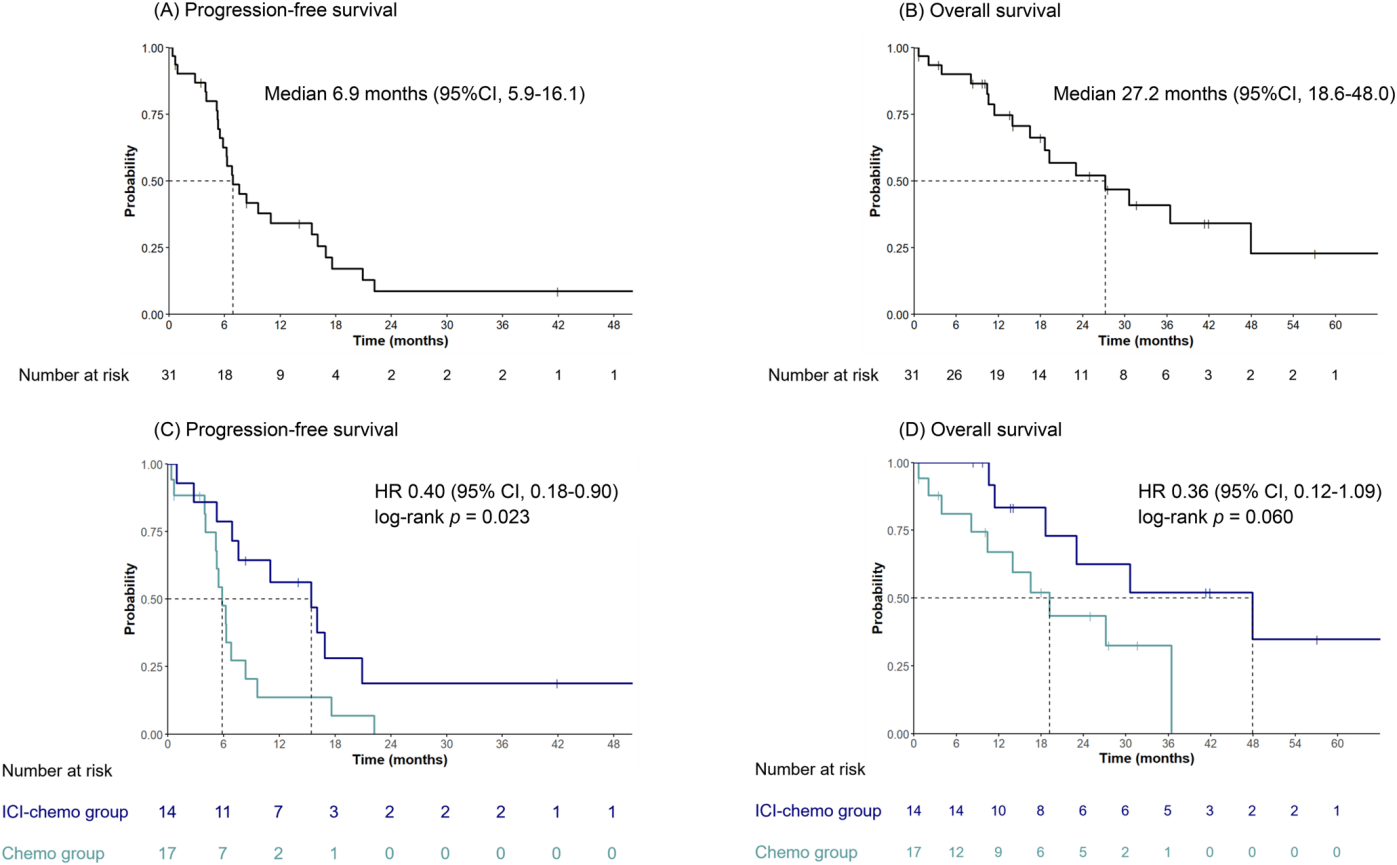
Progression‐free survival (A) and overall survival (B) of the platinum retreatment with or without ICI. Progression‐free survival (C) and overall survival (D) comparing the ICI‐chemo group with the chemo group. ICI, immune checkpoint inhibitor; chemo, chemotherapy.

**FIGURE 3:**
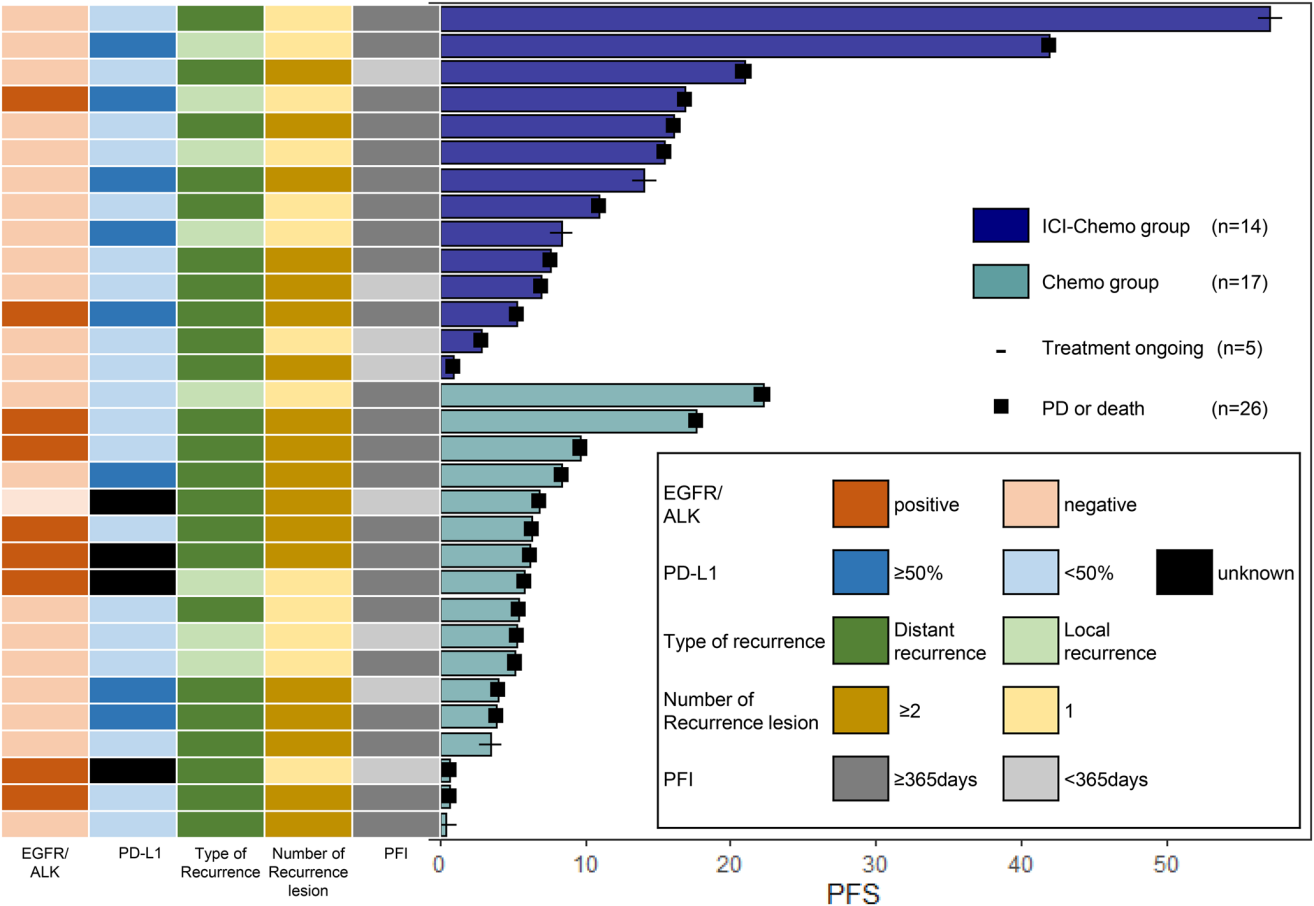
Swimmer plot of progression‐free survival. ICI, immune checkpoint inhibitor; Chemo, chemotherapy; PD, progressive disease; EGFR, epidermal growth factor receptor; ALK, anaplastic lymphoma kinase; PD‐L1, programmed death‐ligand 1; PFI, platinum‐free interval; PFS, progression‐free survival.

At the time of data cutoff (June 30, 2024), the median (range) follow‐up duration for OS was 21.0 (0.69–73.0) months. Of the 31 patients, 43% (6/14) in the ICI‐chemo group and 59% (10/17) in the chemo group had died. The median PFS for platinum retreatment with or without ICI was 6.9 months (95% confidence interval [CI]: 5.9–16.1 months) (Figure 2A). The PFS of the ICI‐chemo group was significantly longer than that of the chemo group (15.4 vs. 5.9 months, log‐rank *p* = 0.023, HR 0.40, 95% CI: 0.18–0.90) (Figure 2C). The PFS rates at 12, 24, 36, 48, and 60 months were 50%, 14%, 14%, 7.1%, and 0%, respectively, in the ICI‐chemo group. In the chemo group, the rates were 12%, 0%, 0%, 0%, and 0%, respectively. The median OS for platinum retreatment with or without ICI was 27.2 months (95% CI: 18.6–48.0 months) (Figure 2B). There was no significant difference in OS between the two groups (47.9 vs. 19.2 months, log‐rank *p* = 0.060, HR 0.36, 95% CI: 0.12–1.09) (Figure 2D). Although this was an exploratory analysis, a trend toward longer PFS with platinum retreatment was observed in patients with local recurrence and a low number of recurrent lesions (Figure 3).

**FIGURE 4:**
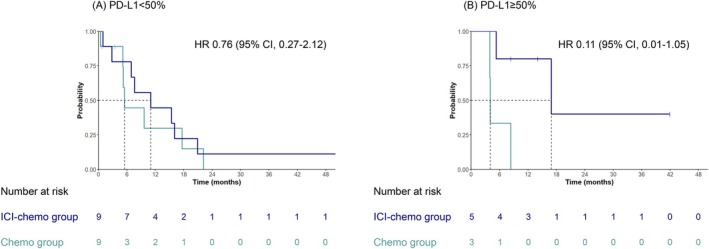
Kaplan–Meier curves of progression‐free survival comparing ICI‐chemo group with chemo group. (A) PD‐L1 < 50% and (B) PD‐L1 ≥ 50%. ICI, immune checkpoint inhibitor; chemo, chemotherapy; PD‐L1, programmed death‐ligand 1.

In patients with PD‐L1 < 50%, the median PFS was 11.0 months in the ICI‐chemo group and 5.5 months in the chemo group (HR, 0.76; 95% CI, 0.27–2.12) (Figure 4A). In patients with PD‐L1 ≥ 50%, the median PFS was 16.9 months in the ICI‐chemo group, showing a trend toward longer PFS compared with 4.0 months in the chemo group (HR, 0.11; 95% CI, 0.01–1.05) (Figure 4B).

Univariate analysis identified platinum retreatment with or without ICI as the only significant predictor of favorable PFS (Table 2). Multivariate analysis showed that platinum retreatment with or without ICI (HR 0.39, 95% CI: 0.18–0.90, log‐rank *p* = 0.027) was the only factor to be significantly associated with longer PFS.

**TABLE 2:** Univariate and multivariate analysis for progression‐free survival after platinum‐based chemotherapy with or without immune‐checkpoint inhibitor.

	Univariate HR (95% CI)	*p*	Multivariate HR (95% CI)	*p*
Age				
< 65/≥ 65 years	1.2 (0.12–12)	0.89	—	—
Sex				
Male/female	1.1 (0.40–2.9)	0.88	—	—
ECOG‐PS				
0/1	1.5 (0.64–3.4)	0.37	—	—
Smoking history				
No/yes	0.96 (0.38–2.4)	0.93	—	—
Histology				
Nonsquamous cell carcinoma/Squamous cell carcinoma	1.0 (0.39–2.7)	0.95	—	—
EGFR/ALK, *n* (%)				
Positive/negative or unknown	1.5 (0.62–3.5)	0.38	—	—
PD‐L1 expression				
≥ 50%/< 50%	0.72 (0.26–2.0)	0.52	—	—
Pathological stage				
II/III	1.4 (0.64–3.1)	0.39	—	—
Type of recurrence				
Local recurrence/distant recurrence	0.54 (0.21–1.4)	0.20	0.53 (0.21–1.4)	0.20
Treatment				
ICI‐chemo/chemo	0.40 (0.17–0.90)	0.027	0.39 (0.18–0.90)	0.027

Abbreviations: ALK, anaplastic lymphoma kinase; ECOG‐PS, Eastern Cooperative Oncology Group performance status; EGFR, epidermal growth factor receptor; PD‐L1, programmed cell death‐ligand 1.

## 4 Discussion

This is thought to be the first report to evaluate the efficacy of adding ICI to platinum retreatment in patients who relapsed after receiving perioperative platinum‐based chemotherapy. We confirmed a long‐term response, a hallmark of ICI therapy, in patients with recurrent NSCLC, as well as in those with advanced NSCLC.

The KEYNOTE‐189 study, which evaluated the efficacy of adding ICI to platinum therapy in advanced NSCLC, reported a significant increase in both PFS and OS in the intention‐to‐treat population (median PFS 9.0 vs. 4.9 months, HR 0.50; median OS 22.0 vs. 10.6 months, HR 0.60) [13]. Our study indicated a prolonged PFS with the addition of ICI, with an HR of 0.40 (95% CI: 0.18–0.90) in the overall population. The combination of ICI with platinum retreatment may be a useful option for postoperative recurrent NSCLC in patients that were previously treated with perioperative platinum‐based chemotherapy, similar to its role in the advanced setting.

PD‐L1 has been utilized as a biomarker to guide treatment strategies for patients with advanced and early‐stage NSCLC [16]. Given the limited sample size, this analysis should be considered exploratory; we found the effect of adding ICI to be particularly pronounced in the PD‐L1 ≥ 50% group, with an HR of 0.11 (95% CI: 0.011–1.1). This is consistent with the results of the KEYNOTE‐189 study, in which higher PD‐L1 expression was associated with greater efficacy of ICI addition on PFS (median PFS 11.1 vs. 4.8 months, HR 0.35; median OS 27.7 vs. 10.1, HR 0.68) [13]. PD‐L1 may be a useful biomarker for guiding the addition of ICI to platinum retreatment, as in advanced‐stage disease.

A post hoc analysis of the KEYNOTE‐189 study revealed that the prolongation of PFS and OS with ICI therapy was more pronounced in patients with intrathoracic‐only recurrence (HR 0.48, 95% CI: 0.32–0.70) than in those with extrathoracic lesions (HR 0.51, 95% CI: 0.41–0.63) [17]. Although this analysis is exploratory and hypothesis‐generating, the swimmer plot in our study showed a trend toward increased PFS in patients with local recurrence and a lower number of recurrent lesions compared with those with distant recurrence. Adding ICI to platinum retreatment may be more effective in patients with local recurrence, fewer lesions, or smaller tumor volumes than in those with distant metastases.

This study has several limitations that warrant mention. First, the cohort size was small and it was derived from a single region, which limits the generalizability of our findings. However, as the number of patients undergoing surgical resection for lung cancer is expected to increase, investigating treatment strategies for postoperative recurrence remains clinically important, and our results may serve as foundational data for future studies. Second, the retrospective nature of the study may also have introduced patient selection bias. At the participating institutions, perioperative chemotherapy was generally administered to patients with a performance status of 0–1, creatinine clearance ≥ 60 mL/min, and no significant cardiac dysfunction. In addition, ICI were not administered in combination with platinum retreatment in patients with comorbid interstitial lung disease or autoimmune disorders. Third, because this study spans a 10‐year period, changes in standard surgical procedures and postrecurrence systemic therapies over time may have influenced the treatment outcomes. Conversely, a key strength of this study is its focus on evaluating the impact of adding ICI to platinum retreatment for postoperative recurrence following perioperative therapy, which is investigated here for the first time.

## 5 Conclusions

Adding ICI to platinum retreatment may be an effective treatment option for patients who relapsed after receiving perioperative platinum‐based chemotherapy. This approach may be especially beneficial for patients with PD‐L1 expression ≥ 50%. Larger prospective studies are warranted to validate these findings.

## Author Contributions

**Toshiaki Takakura:** writing – original draft, investigation, data curation. **Ryota Shibaki:** conceptualization, writing – review and editing, supervision. **Yuhei Harutani:** data curation, investigation. **Yusuke Murakami:** data curation, investigation. **Atsushi Washioka:** investigation, data curation. **Hiroaki Akamatsu:** data curation, investigation.**Nobuyuki Yamamoto:** data curation, investigation.

## Funding

Toshiaki Takakura, Atsushi Washioka, Yusuke Murakami, Yuhei Harutani declare they have no financial interests. Ryota Shibaki has received honoraria from AstraZeneca K.K., Chugai Pharmaceutical Co. Ltd., MSD K.K., Taiho Pharmaceutical Co. Ltd., Ono Pharmaceutical Co. Ltd., Daiichi Sankyo Co. Ltd., Boehringer Ingelheim Japan Inc. Hiroaki Akamatsu has received honoraria from AstraZeneca K.K., Chugai Pharmaceutical Co. Ltd., MSD K.K., Ono Pharmaceutical Co., GlaxoSmithKline K.K.; committee member in patient advocacy committee of international association for the study of lung cancer. Nobuyuki Yamamoto has received grants from PRiME‐R Inc., Amgen K.K., EPS Co. Ltd., ONO PHARMACEUTICAL CO. LTD., GlaxoSmithKline K.K., CHUGAI PHARMACEUTICAL CO. LTD., Eli Lilly Japan K.K., Novartis Japan., Pfizer R&D Japan, Medpace Japan K.K., Janssen Pharmaceutical K.K., IQVIA Solutions Japan G.K., AstraZeneca K.K., AbbVie Inc., A2 Healthcare Corporation, Kyowa Kirin Co. Ltd., TAIHO PHARMACEUTICAL, CO. LTD., SYNEOS HEALTH CLINICAL K.K., Nippon Boehringer Ingelheim Co. Ltd., Nippon Chemical Industrial Co. Ltd., Bristol Myers Squibb, Medical Market Vision Co. Ltd., RPM Co., Merck Sharp & Dohme; honoraria from AstraZeneca K.K., Accuray Japan K.K., Otsuka Pharmaceutical Co. Ltd., Guardant Health Japan Corp., KYORIN Pharmaceutical Co. Ltd., DAIICHI SANKYO, INC., Takeda Pharmaceutical Co. Ltd., Tsumura & Co., Eli Lilly Japan K.K., Novartis Japan., Novocure Ltd., Miyarisan Pharmaceutical Co. Ltd., Medpeer Inc., USACO Corporation, Amgen K.K., AbbVie Inc., ONO PHARMACEUTICAL CO. LTD., Kyowa Kirin Co. Ltd., GlaxoSmithKline K.K., TAIHO PHARMACEUTICAL, CO. LTD., CHUGAI PHARMACEUTICAL CO. LTD., Terumo.co.jp., Nippon Boehringer Ingelheim Co. Ltd., Nippon Chemical Industrial Co. Ltd., Pfizer Japan., Merck Biopharma Co. Ltd., Janssen Pharmaceutical K.K., Merck Sharp & Dohme.

## Ethics Statement

This study was performed in line with the principles of the Declaration of Helsinki. Approval was granted by the Wakayama Medical University Ethics Committee (approval number: 4408).

## Consent

Informed consent was obtained from all individual participants included in the study. All authors consent for publication.

## Conflicts of Interest

The authors declare no conflicts of interest.

## Supporting information


**Table S1:** Regimens of platinum retreatment.

## Data Availability

The datasets generated during and/or analyzed during the current study are available from the corresponding author on reasonable request.
