# Supplementary material for: Addition of Immune Checkpoint Inhibitor to Platinum Retreatment for Recurrent Non‐Small Cell Lung Cancer After Perioperative Chemotherapy: A Multicenter Retrospective Study
Source: Thorac Cancer. 2026 Mar 30;17(7):e70269. doi: 10.1111/1759-7714.70269 (PMC13070734; doi:10.1111/1759-7714.70269)
Supplement: Supplementary file 1 — Table S1: Regimens of platinum retreatment. [file TCA-17-e70269-s001.docx]

Table S1. Regimens of platinum retreatment

| Regimens | Number of patients |
| --- | --- |
| Carboplatin + Pemetrexed | 7 |
| Carboplatin + Pemetrexed + Bevacizumab | 2 |
| Carboplatin + nab-Paclitaxel | 5 |
| Carboplatin + Paclitaxel | 2 |
| Cisplatin + Pemetrexed | 1 |
| Carboplatin + Pemetrexed + Pembrolizumab | 4 |
| Carboplatin + Pemetrexed + Ipilimumab + Nivolumab | 1 |
| Carboplatin + nab- Paclitaxel + Pembrolizumab | 2 |
| Carboplatin + Paclitaxel + Pembrolizumab | 1 |
| Carboplatin + Paclitaxel + Bevacizumab + Atezolizumab | 6 |
